# *In silico* Screening and Behavioral Validation of a Novel Peptide, LCGA-17, With Anxiolytic-Like Properties

**DOI:** 10.3389/fnins.2021.705590

**Published:** 2021-08-02

**Authors:** Anton V. Malyshev, Iuliia A. Sukhanova, Alexander S. Zlobin, Vasilina R. Gedzun, Vsevolod V. Pavshintsev, Ekaterina V. Vasileva, Arthur O. Zalevsky, Igor I. Doronin, Nikita A. Mitkin, Andrey V. Golovin, Maxim L. Lovat, Georgy I. Kovalev, Yurii A. Zolotarev, Askar R. Kuchumov, Gennady A. Babkin, Bernhard Luscher

**Affiliations:** ^1^Lactocore, Inc., Plymouth, MI, United States; ^2^Shemyakin and Ovchinnikov Institute of Bioorganic Chemistry of the Russian Academy of Sciences, Moscow, Russia; ^3^Faculty of Bioengineering and Bioinformatics, Lomonosov Moscow State University, Moscow, Russia; ^4^Sirius University of Science and Technology, Sochi, Russia; ^5^Department of Human and Animal Physiology, Faculty of Biology, Lomonosov Moscow State University, Moscow, Russia; ^6^Federal State Budgetary Institution, Research Zakusov Institute of Pharmacology, Moscow, Russia; ^7^Institute of Molecular Genetics of National Research Centre “Kurchatov Institute”, Moscow, Russia; ^8^Department of Biology, The Pennsylvania State University, University Park, PA, United States; ^9^Department of Biochemistry and Molecular Biology, The Pennsylvania State University, University Park, PA, United States

**Keywords:** peptide drugs, drug screening, novel ligand, NMDA receptor, alpha(2)delta subunit, computational approach

## Abstract

The aim of the study was to develop better anxiolytics and antidepressants. We focused on GABA_*A*_ receptors and the α2δ auxiliary subunit of V-gated Ca^2+^ channels as putative targets because they are established as mediators of efficacious anxiolytics, antidepressants, and anticonvulsants. We further focused on short peptides as candidate ligands because of their high safety and tolerability profiles. We employed a structural bioinformatics approach to develop novel tetrapeptides with predicted affinity to GABA_*A*_ receptors and α2δ. *In silico* docking studies of one of these peptides, LCGA-17, showed a high binding score for both GABA_*A*_ receptors and α2δ, combined with anxiolytic-like properties in a *Danio rerio* behavioral screen. LCGA-17 showed anxiolytic-like effects in the novel tank test, the light–dark box, and the social preference test, with efficacy comparable to fluvoxamine and diazepam. In binding assays using rat brain membranes, [^3^H]-LCGA-17 was competed more effectively by gabapentinoid ligands of α2δ than ligands of GABA_*A*_ receptors, suggesting that α2δ represents a likely target for LCGA-17. [^3^H]-LCGA-17 binding to brain lysates was unaffected by competition with ligands for GABA_B_, glutamate, dopamine, serotonin, and other receptors, suggesting specific interaction with α2δ. Dose-finding studies in mice using acute administration of LCGA-17 (i.p.) demonstrated anxiolytic-like effects in the open field test, elevated plus maze, and marble burying tests, as well as antidepressant-like properties in the forced swim test. The anxiolytic effects were effectively blocked by bicuculline. Therefore, LCGA-17 is a novel candidate anxiolytic and antidepressant that may act through α2δ, with possible synergism by GABA_*A*_ receptors.

## Introduction

Recent decades have shown a steadily increased interest in peptides as pharmacological therapeutics. Advances in structural biology have helped to broaden the allure of peptides beyond the treatment of hormonal deficiencies. Chemically, peptides represent a unique class of compounds positioned in between small molecules and proteins yet biochemically and therapeutically distinct from both. The structural versatility of peptides together with their superior safety and tolerability profiles make them promising drug candidates (Lau and Dunn, 2018). Here we explored the utility of peptides as novel anxiolytics and putative antidepressants, focusing on A-type receptors for γ-aminobutyric acid (GABA_*A*_Rs) and the α2δ subunit of voltage-gated Ca^2+^ channels (VGCC) as potential targets. GABA_*A*_Rs are GABA-gated chloride channels that serve as the principal mediators of neural inhibition in the brain, and they are known as the targets of classical anxiolytics including the benzodiazepines (BZD) and barbiturates (Möhler, 2012), as well as the antidepressant brexanolone, an intravenous preparation of the endogenous neurosteroid, allopregnanolone (Lüscher and Möhler, 2019). These agents act as positive allosteric modulators (PAMs) of GABA_*A*_Rs, through distinct ligand binding sites. The α2δ subunit is structurally and functionally distinct from GABA_*A*_Rs; it acts to increase the functional expression of not only VGCC but also NMDA receptors and possibly other ion channels (Hendrich et al., 2008; Yang et al., 2009; Tae et al., 2017; Chen et al., 2018). Notably, similar to PAMs of GABA_*A*_Rs, inhibitory ligands of α2δ such as pregabalin and gabapentin (gabapentinoids) are widely used as anxiolytics and anticonvulsants and also as antinociceptives (Bandelow et al., 2008; Baldwin et al., 2011; Ahmed et al., 2019). Interestingly, recent evidence suggests that the anxiolytic (but not antinociceptive) effects of gabapentinoids involve enhanced GABAergic transmission (Yu et al., 2019). Moreover, and consistent with their highly overlapping therapeutic indications, GABA_*A*_Rs, and VGCCs may also serve as shared targets of the prototypical antidepressant, fluoxetine (Robinson et al., 2003; Ye et al., 2008; Normann et al., 2018).

Novel computational approaches allow for an *in silico* search for peptide candidates that bind to specific sites of a receptor (Farhadi and Hashemian, 2018; Zalevsky et al., 2019). GABA_*A*_Rs and the α2δ auxiliary subunit of VGCCs and NMDA receptors are of interest both through their overlapping therapeutic potential and because they are established as targets of diverse endogenous and exogenous peptide modulators. For example, α-casozepine is a biologically active peptide from bovine milk hydrolysate with affinity to GABA_*A*_Rs and anxiolytic-like properties *in vivo* (Miclo et al., 2001). Thrombospondins are endogenous, astrocyte-derived extracellular matrix proteins that interact with α2δ to promote the formation of new synapses (Christopherson et al., 2005; Lau et al., 2017). A wide variety of peptide toxins and synthetic peptide mimetics targeting VGCCs have been discovered (Yamamoto and Takahara, 2009; Vink and Alewood, 2012).

We here employed computational modeling to search for peptides that interact with GABA_*A*_Rs and the α2δ subunit of VGCCs, combined with high throughput behavioral screening of candidate peptides in *Danio rerio*, to search for novel anxiolytics and potential antidepressants. We identified the tetrapeptide FQSE (from here onwards referred to as LCGA-17) as an anxiolytic-like ligand of VGCCs, with behavioral efficacy potentially facilitated by low-affinity interaction with the pregnanolone (PREGS) binding site of GABA_*A*_Rs. Radioligand binding assays using rat brain extracts confirmed that LCGA-17 was efficiently and specifically competed by gabapentin. Further behavioral characterization of LCGA-17 in mice confirmed that LCGA-17 has therapeutic potential as a rapid acting anxiolytic and possible antidepressant also in mammals.

## Materials and Methods

### Animals

All animal experiments were conducted in accordance with the European Directive 2010/63/EU of the European Parliament (Council of 22 September 2010 on the protection of animals used for scientific purposes) and the Russian “GOST 33216-2014” Guidelines for the maintenance and care of laboratory animals and were approved by the local Bioethics Commission LLC of the Institute of Mitoengineering of MSU. Wildtype *D. rerio* (246 animals, shortfin phenotype, 6–8 months of age, male to female ratio 50:50) were kept in a ZebTEC (Tecniplast S.p.A, Buguggiate, Italy) recirculating system, and housed under a 14/10-h light/dark cycle (8 AM “on” and 10 PM “off”). The system parameters were maintained automatically with water set at 28°C, pH 6.8–7.5, 550–700 mOsm/L and constant aeration. Feeding was carried out twice a day with special food for fish (special diet services, scientific fish food, SDS 300–400). The mice (BALB/c, 6–8 week-old males) were obtained from the nursery for laboratory animals “Pushchino,” Russia. They were housed under a 12 h dark/light cycle in a temperature-controlled environment, with food and water *ad libitum*.

### Drug Treatment and Behavioral Screening in *Danio rerio*

Fluvoxamine (flv, 5 and 10 mg/kg, in saline) and diazepam (diaz, 1.25 and 5 mg/kg, in 0.3% propylene glycol, 0.06% ethanol) were purchased from Sigma-Aldrich Inc., and administered in a volume of 10 μl. Control groups were treated in parallel with vehicle alone. For drug injection, the fish were anesthetized briefly by placing them in 10°C water. The vehicle controls were tested in parallel. LCGA peptides (supplied by Lactocore, Inc., 98% purity) were administered at a dose of 1 and 10 mg/kg (i.p., prepared in saline on the day of the experiment). The peptides had the following amino acid composition: LCGA-17 (FQSE = PheGlnSerGlu); LCGA-26 (DKTE = AspLysThrGlu); LCGA-59 (WDQV = TrpAspGlnVal) and LCGA-83 peptide (FLPY = PheLeuProTyr).

Behavioral testing was initiated 10 min after drug injection, starting with the novel tank test (NTT), followed immediately thereafter by the light–dark box (LDB) and the social preference test (SPT). The NTT was adapted from Maximino et al. (2013) with the behavior video-recorded and the data processed using EthoVision XT14 (Noldus). The distance traveled, speed, number of visits to the bottom, middle and top thirds of the aquarium, respectively, and the times spent in each of these zones during the initial 5 min in the tank were recorded. A decreased time spent at the surface of the NTT apparatus reflects a reduction in exploratory behavior or increased hiding motivation (Sackerman et al., 2010). The behavior in the LDB (Maximino et al., 2011) and SPT (Parker et al., 2014) was video recorded and processed using RealTimer (Open Science LCC, Russia). The fish were added to the center zone of a three-zone aquarium and allowed to adapt for 1–2 min before removal of the septa separating the center from the flanking zones. The time spent and the number of visits to the light and dark flanking zones and the latency to enter the lit zone were recorded during a 5-min session. Stress of fish is associated with increased time spent in the dark compartment (scototaxis) (Maximino et al., 2011). For the SPT, the residence time and the number of visits to the three equally sized zones (near the shoal, in the center zone of the aquarium, and near the wall opposite to the shoal) were assessed during a 5-min session. Increased shoal preference (shoaling reflex) is thought to represent a protective response to a predator (Nguyen et al., 2014). All behavioral testing was done in the light phase using 500 lux (NTT, LDB) or 200 lux (SPT) illumination.

### Drug Treatment and Behavioral Characterization in Mice

The mice were injected (i.p.) with LCGA-17 (in saline), diazepam (in 0.3% propylene glycol, 0.06% ethanol), bicuculline (in saline) or with vehicle alone, 30 min before testing and at the dosages indicated in the Figure legends. The open field test (OFT) was used to assess locomotion. The total distance traveled in a 5 min session was recorded using Ethovision XT14 (Prut and Belzung, 2003). The elevated plus maze (EPM) and the marble burying test (MBT) were used to assess anxiolytic-like drug activity. For the EPM (Pellow et al., 1985), the percentage of time spent in the open arms, the percentage of open arm entries, and the number of total arm entries during a 5-min session were recorded using Ethovision. “Anxiety Index” (AI), an integrative behavioral measure in the EPM was calculated as follows: 100 × {1 − [(time spent on open arms/total time on the maze)/2 + (number of entries to the open arms/total exploration on the maze)/2]} (Cohen et al., 2011). The MBT (Njung’e and Handley, 1991) was carried out in a 19 × 29 × 13 cm cage filled 5 cm deep with wood-chip bedding. Twenty marbles (5.2 g, 15 mm diameter, randomly colored) were evenly placed on the surface of the bedding in four rows of five pieces. At the end of a 30 min test, the animal was removed, and the marbles buried in bedding by at least two-thirds were recorded. The number of marbles buried in this test is reduced by anxiolytics and antidepressants such as BZDs and selective serotonin reuptake inhibitors (SSRIs), respectively (Takeuchi et al., 2002; Nicolas et al., 2006). The forced swim test (FST) was used for a preliminary assessment of antidepressant drug activity as described (Colelli et al., 2014). Briefly, on day one the mice were exposed to a 10 min pre-swim in a cylinder filled with 24 ± 1°C water. The next day they were re-exposed to the same apparatus for a 5 min test session with the behavior video recorded. The total time spent immobile was quantitated off-line visually. The interval between sequential behavioral tests was 2–3 days with the sequence OFT, EPM, MBT, and FST. All the testing and data analyses were performed by experimenters blind to treatment.

### Computational Studies

We used liquid chromatography-tandem mass spectrometry to separate *Bos taurus* milk hydrolysates (supplied by Lactocore, Inc.) into protein fractions. The major peptides of these fractions were identified and computationally converted into 288 unique tetrapeptides, using a sliding window with a step of one amino acid. We first performed blind docking of all 288 tetrapeptides to the α1β2γ2 GABA_*A*_R, using the atomic receptor models 6 × 3S, 6 × 3T, 6 × 3U, 6 × 3V, 6 × 3W, 6 × 3X, 6 × 3Z, and 6 × 40 as templates (Kim et al., 2020) and QuickVina2.1 software (Alhossary et al., 2015) with modifications of Autodock Vina (Hassan et al., 2017). Prior to running the docking algorithm, all the receptor structures were stripped of co-crystallized modulators and antibodies. The box used for docking was set to include the entire receptor molecule. A transmembrane site of interest was identified visually (Supplementary Figure 1A). The same strategy was used for blind docking of allopregnanolone and pregnenolone sulfate (PREGS) to the GABA_*A*_R and identification of a putative neurosteroid binding pocket (Supplementary Figures 1B,C). For subsequent peptide docking runs we used the proprietary Peptimize algorithm (Lactocore, Inc.), based on the Peptogrid algorithm (Zalevsky et al., 2019). The docking box for this run was designed as a bounding box for PREGS moieties and extended to account for the larger size of tetrapeptides compared to neurosteroids (Supplementary Figure 1C).

A α2δ-1-peptide interaction site was identified analogously. The 6JPA model of α2δ-1 was subjected to homology modeling with SwissModel to reconstruct the missing regions Thr831-Cys842 and Pro913-Met972 of the VGCC structure (Zhao et al., 2019). In particular, the second of these domains is large and the structure unknown. To account for all its possible structures we performed a molecular dynamic (MD) simulation of 1 μs of the whole α2δ-1 domain as described in Supplementary Data 1. We monitored the reconstructed loop’s root mean square deviation (RMSD) as a subjective measure of its stabilization. We observed that the system is mostly stabilized by 700 ns and picked this frame as a reference model for subsequent docking studies (Supplementary Figure 2). Gabapentin was blindly docked into the whole receptor, and the best pose was utilized to highlight the site for targeting. In search of additional support for this site, we extracted the frames from the trajectory every 100 ns and subjected them to blind docking. We obtained solid support for the identified site as most of the best gabapentin poses were spatially clustered within that site. Analogous to the GABA_*A*_ receptor study, the box for peptide docking to the α2δ-1 subunit encompassing the gabapentin site was defined (Supplementary Figure 3C). We note that the reconstructed Pro913-Met972 region is far removed from the optimal gabapentin binding site and therefore unlikely to influence the binding site selection process.

Next, the results of docking the peptides against the 6 × 3S GABA_*A*_R model and against the 700 ns frame of the MD trajectory of VGCCs were rescored using Peptimize algorithm. To identify the most accurate interaction profiles of the peptides with the two receptors, we performed a redocking round with Autodock Vina (Trott and Olson, 2009) with the exhaustiveness parameter set to 512 (Rentzsch and Renard, 2015). The best peptide poses for each site were then subjected to 100 ns atomic MD simulations to equilibrate the peptide in its binding site and further refine the interaction profile while also taking water molecules into account (see MD protocol in Supplementary Data 1).

### Radioligand Binding Assay

[^3^H]-LCGA-17 (70 Ci/mmol) was synthesized by high-temperature solid-state catalytic isotope exchange (HSCIE) by A. Yu. Zolotarev at the Department of Physiologically Active Substances Chemistry, Institute of Molecular Genetics of RAS, Moscow, Russia (Zolotarev et al., 2003). Briefly, rat brain tissue was homogenized in 25 volumes of cold 50 mM Tris–HCl, pH 7.4 using a Teflon-glass homogenizer. The homogenate was centrifuged (20 min, 40,000 × g) and the precipitated membranes resuspended in the same buffer and centrifuged twice more. The pellet was resuspended in 15 mL of the same buffer and used for the radioligand analysis. Binding reactions (500 μL) contained 250 μL of the membrane suspension, 50 μL of [^3^H]-LCGA-17 (70 Ci/mmol, final concentration 16 nM) and 200 μL of 50 mM Tris–HCl buffer (pH = 7.0). The reaction was incubated at room temperature for 25 min, filtered through GF/B filters (Whatman) preconditioned with 0.3% polyethyleneimine, and the filters washed and air-dried, and the bound [^3^H] activity counted in a Tri-Carb 2900TR counter (Perkin Elmer). The IC_50_ of putative competitor compounds was determined by supplementing binding reactions with corresponding test compounds at 10^–10^ to 10^–4^ M. The list of compounds tested is available in Supplementary Table 1.

### Statistical Analyses

Statistical analyses were performed using Prism 9.0 (GraphPad). Normality of distributions was assessed using Shapiro–Wilk’s W. Normally distributed data were analyzed by one-way ANOVA and Dunnett’s *post hoc* test. Non-normally distributed data were analyzed by Kruskal–Wallis followed by Dunn’s *post hoc* test. The data are presented as box and whisker plots. For pairwise comparisons (*in vivo* screening in *D. rerio*), we used Mann–Whitney (M–W) *U*-test. False discovery rate (FDR) correction using Benjamini and Hochberg (1995) was applied, with a significance threshold set of *q* = 0.05.

## Results

### Identification of LCGA-17 Binding Sites to GABA_*A*_ Receptor and the α2δ Subunit of VGCC by Computational Modeling

The objective of this study was to explore the suitability of milk-hydrolysate-derived peptides as a source of novel central nervous system (CNS)-active therapeutics, with a special focus on neuronal protein targets that are known to mediate the effects of clinically successful anxiolytics. To begin we performed *in silico* blind docking experiments with 288 milk hydrolysate-derived tetrapeptides to map a potentially druggable space on the entire α1β2γ2 GABA_*A*_R structure (Supplementary Figure 1A). We then discarded all positional clusters situated on the exterior face of the transmembrane domain to avoid potential complications for peptides to enter and equilibrate within the membrane. We also excluded the BZD, ketamine, flurazepam, bromine, and barium binding sites, since therapeutic ligands for these sites have already been described (Puthenkalam et al., 2016). We then focused our attention on an internal transmembrane binding site, which we hypothesized may represent a binding site for PREGS and allopregnanolone. We found that allopregnanolone preferentially binds the external face of a transmembrane region in several distinct sites including ones recently identified for α1β3γ2 (Supplementary Figure 1B; Chen et al., 2019). Almost all of the best PREGS docking poses mapped to the transmembrane domain of the channel, which corresponded perfectly with the same site selected by modeling of peptide docking (Supplementary Figure 1C). Thus, a peptide modulator under development was provisioned to act as a functional analog of PREGS.

In addition to GABA_*A*_Rs, we scored all the hydrolysate-derived peptides for binding to the α2δ subunit of VGCC, exploring the possibility of candidates with high docking scores for both targets. In particular, we discovered a peptide docking site on α2δ that we had previously identified as a putative binding site for the α2δ ligand, gabapentin, using a blind docking experiment (Supplementary Figure 3). The two Peptimize scores from docking studies with the GABA_*A*_R and α2δ were averaged to produce a unified binding score for each peptide. This score was then used to rank the hydrolysate-derived peptides by predicted affinity to both targets. The top three peptides identified in this way were HKEM, FFVA, and FQSE. Of these, the HKEM peptide was discarded due to a high probability of methionine oxidation that predicts peptide toxicity (Schöneich, 2005). The FFVA peptide was discarded due to low predicted solubility. The FQSE peptide (from here onwards named LCGA-17) and three lower-ranking control peptides (DKTE, WDQV, and FLPY, in the following referred to as LCGA-26, -59, and -83, respectively) were selected for further validation.

**FIGURE 1:**
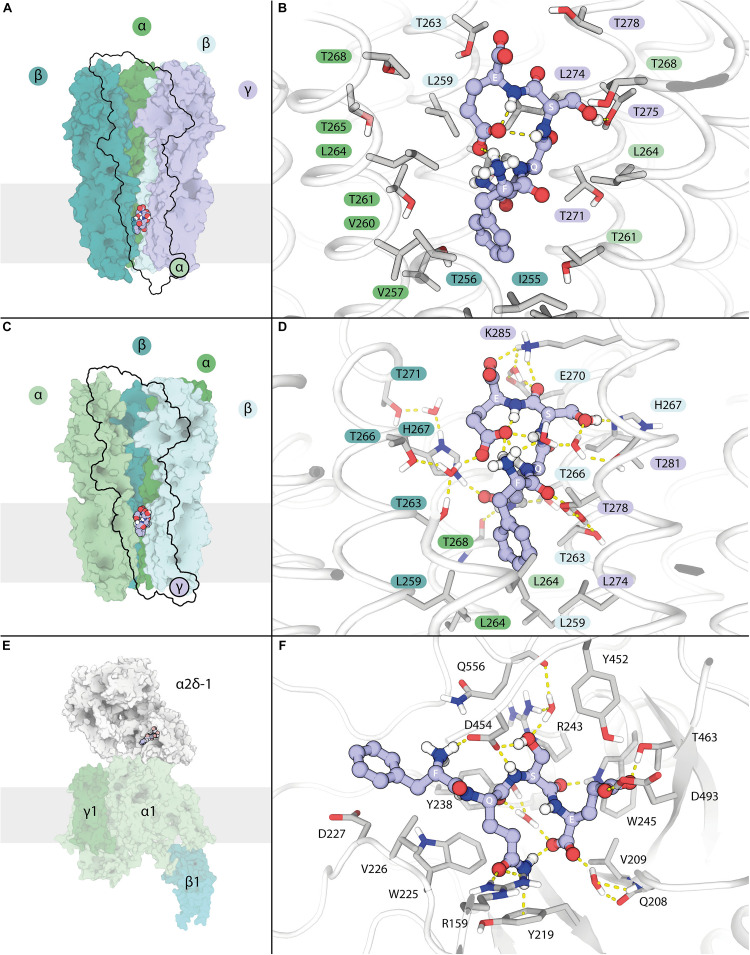
Interactions of LCGA-17 with GABA_*A*_ receptors and α2δ of VGCCs revealed by modeling of binding poses. **(A)** Low magnification view of a “wide-lumen” α1β2γ2 GABA_*A*_R with bound LCGA-17 peptide depicted in spheres. The proximal α1 subunit is omitted for clarity. **(B)** Major interactions of LCGA-17 with its lower GABA_*A*_R transmembrane binding site. **(C)** Low magnification view of a “narrow-lumen” α1β2γ2 GABA_*A*_R with bound LCGA-17. The γ2 subunit is omitted for clarity. **(D)** Major interactions of LCGA-17 in the upper GABA_*A*_R transmembrane binding pocket. The colors of amino acid labels match the color code of subunits in panels **(A,C)**. **(E)** Overview of VGCC with LCGA-17 bound to the α2δ-1 subunit binding pocket. **(F)** Major interactions of LCGA-17 within the α2δ-1 binding pocket. Note that non-polar hydrogen atoms are omitted for clarity in all images. Hydrogen bonds are shown as dashed lines. Carbon atoms of LCGA-17 are shown in purple and gray, oxygen in red; nitrogen in blue.

Possible atomic interactions of LCGA-17 with its α2δ binding domain were investigated in greater detail using a redocking round with enhanced accuracy followed by a 100 ns molecular dynamics study, to account for potential solvation effects and assess the overall short-term stability of particular binding poses (Figures 1A–E and Supplementary Videos 1–3). The width of the transmembrane channel pore varies among different GABA_*A*_R crystal structures (Kim et al., 2020), leading us to compare the binding poses of LCGA-17 in GABA_*A*_R structures with both a narrow (6 × 3S) and wider channel pore (6 × 3W). The initial binding pose of LCGA-17 in the structure 6 × 3W resembled the binding pose of PREGS in α2δ. However, when simulating receptor-ligand complex movement by MD, we found that the conformation of LCGA-17 in this structure was unconstrained and highly dynamic. The peptide had rapidly adopted a distinctive “enclosed” structure characterized almost exclusively by intra- rather than intermolecular interactions (Figures 1A,B and Supplementary Video 1). Indeed, attractive interactions between LCGA-17 and the receptor were largely absent, suggesting shape complementarity as the only evidence in support of interaction in the “wider-pore” case. Autodock Vina in this case predicted a binding energy of −7.0 kcal/mol. The same enclosed peptide structure was observed as an initial binding pose for the narrower channel conformation of the 6 × 3S receptor structure, although this pose was located more toward the extracellular portion of the channel. In this pose, the peptide conformation was remarkably stable, involving numerous interactions with the receptor (Figures 1C,D and Supplementary Video 2), which was also reflected in a greater binding energy (−7.8 kcal/mol). Comparing the interaction profiles, we propose that this second binding mode represents a possible functional form of LCGA-17 when bound to GABA_*A*_Rs. The binding mode of LCGA-17 in the α2δ site was dynamically stable (Supplementary Video 3) and supported by an extensive network of stable, attractive interactions within the α2δ binding pocket depicted in Figure 1F and is reflected in a high predicted binding energy of −8.5 kcal/mol. Notably and in contrast to the conformation of LCGA-17 in the GABA_*A*_R PREGS binding pocket, LCGA-17 did not fold into an enclosed conformation when bound to α2δ (Figures 1E,F). Collectively, these studies identify LCGA-17 as a promising ligand for α2δ, with additional potential for binding to the GABA_*A*_R PREGS binding pocket.

### Behavioral Screening of LCGA Peptides in *Danio rerio*

**FIGURE 2:**
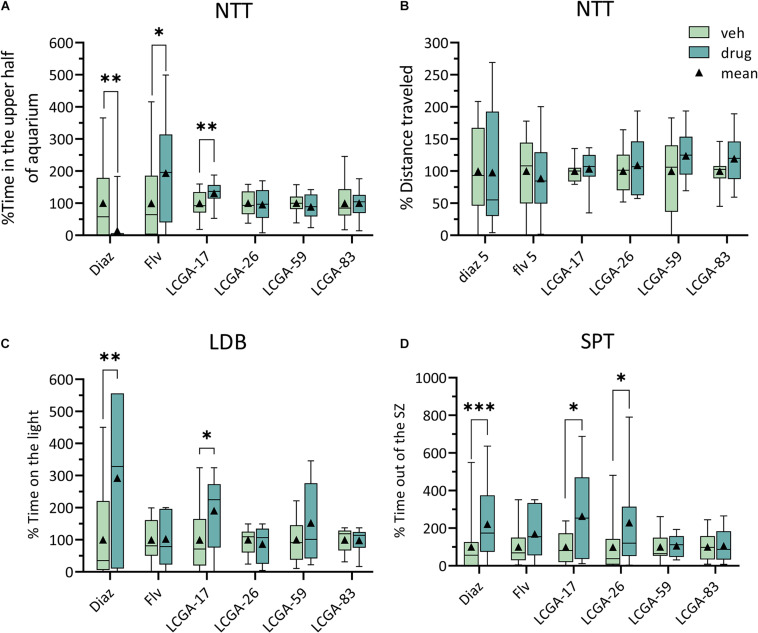
Behavioral analyses of the anxiolytic-like properties of LCGA tetra-peptides in *Danio rerio*. **(A,B)** In the NTT, LCGA-17 (10 mg/kg) increased the time the fish spent in the upper half of the tank, similar to fluvoxamine (Flv, 5 mg/kg), indicating an anxiolytic-like response **(A)**. By contrast, diazepam (Diaz, 5 mg/kg) reduced the time spent in the upper half of the tank **(A)**. None of the treatments affected the distance traveled **(B)**. **(C)** In the LDB, LCGA-17 increased the time spent in the lit compartment, closely mimicking the anxiolytic-like effect of diazepam. **(D)** In the SPT, both LGCA-17 and LCGA-26 increased the time spent out of the shoaling zone, again mimicking an anxiolytic-like effect of diazepam. Neither fluvoxamine (5 mg/kg) nor LCGA-59 and LCGA-83 (10 mg/kg) affected the behavior in the LDB and SPT **(B,C)**. Data are presented as % of vehicle control. ^∗^*p* < 0.05, ^∗∗^*p* < 0.01, ^∗∗∗^*p* ≤ 0.001, *n* = 18–30, Mann–Whitney, with corrections for multiple comparisons using the FDR method, at a *q* threshold of 0.05.

To explore the potential of LCGA-17 and three other LCGA peptides as behaviorally active anxiolytics we first employed high throughput behavioral tests in *D. rerio*, using diazepam and fluvoxamine as active control compounds with established anxiolytic efficacy in patients. The three tests employed have in common that they examined the fishes’ resolve to overcome different naturally aversive conditions such as novelty (NTT), bright light (LDB), or moving away from the shoal (SPT). In previous characterizations of diazepam and fluvoxamine using these tests, both compounds affected mainly the time spent in the upper half of the tank (NTT), the time in the light (LDB), and the time away from the “shoal” (SPT), respectively (data not shown), which led us to focus on these three parameters as the most informative. Each of the six compounds was tested at two dosages with the summary statistics of the most informative dosages depicted in Figure 2. For a summary of all the dosages tested see Supplementary Table 2.

In the NTT, LCGA-17 increased the time spent in the upper half of the tank, thereby mimicking the anxiolytic-like effect of fluvoxamine (Figure 2A) (for statistics see Figure legend). None of the other LCGA peptides had a measurable effect in this test. However, diazepam (5 mg/kg) reduced the time spent in the upper half of the tank. This drug effect did not represent sedation as the total distance traveled remained unaffected (Figure 2B). In the LDB, LCGA-17 increased the time spent in the lit compartment, thereby reproducing the anxiolytic-like effect of diazepam (Figure 2C). Neither fluvoxamine nor the other LCGA peptides affected the behavior in this test. In the SPT, both LCGA-17 and LCGA-26 increased the time the fish spent out of the shoaling zone, thereby reproducing the anxiolytic-like effect of diazepam (Figure 2D). However, fluvoxamine, LCGA-59, and LCGA-83 failed to affect behavior in this test. Collectively, these experiments predict that LCGA-17 has anxiolytic activity, with an efficacy comparable to diazepam and fluvoxamine.

### Radioligand Binding Assays of LCGA-17 in Brain Lysates

**FIGURE 3:**
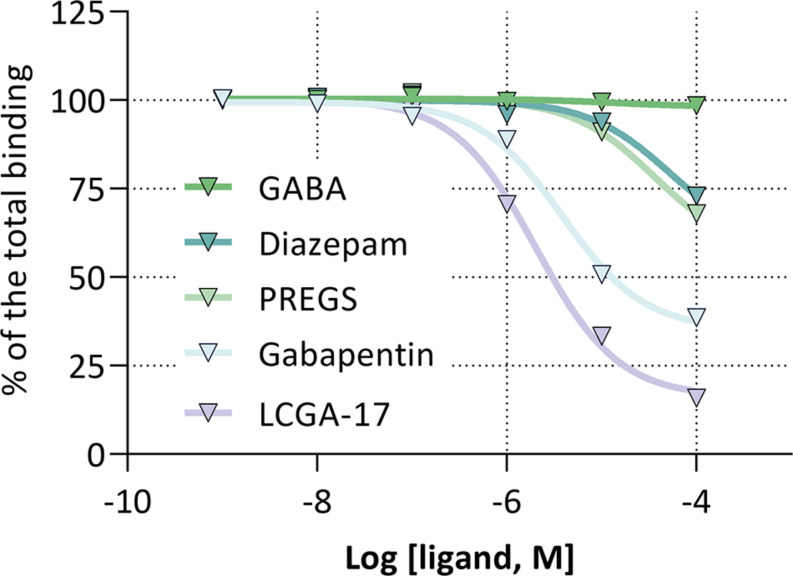
Competition of [^3^H]-LCGA-17 binding to cortical membranes by high-affinity ligands of the GABA_*A*_R BZD and neurosteroid binding sites and the gabapentinoid binding pocket of α2δ. The fraction of [^3^H]-LCGA-17 bound is plotted as a function of the log (ligand, M). IC_50_: gabapentin, ∼11 μM; LCGA-17, ∼2 μM; GABA, diazepam, and PREGS, >100 μM.

Molecular modeling of LCGA-17 binding to GABA_*A*_R and α2δ suggested the presence of distinctive binding sites. In support of this notion, radioligand binding of [^3^H]-LCGA-17 to rat brain membranes and competition with unlabeled LCGA-17 revealed an IC_50_ of ∼2 μM. Gabapentin showed similar affinity (IC_50_ ∼ 11 μM) for the same site, while diazepam and PREGS were ineffective as competitors (IC_50_ > 100 μM) (Figure 3). Moreover, we detected no cross-reactivity with other GABA_*A*_R binding sites (GABA, muscimol, bicuculline, gabazine, CGS-9895), nor with ligand binding sites of other major neurotransmitter receptors (GABA_B_: baclofen; dopamine receptors: haloperidol, sulpiride, spiperone, 7-OH-DPAT; serotonin receptors: ketanserin; acetylcholine receptors: nicotine; and glutamate receptors: glutamate, glycine, Ro-256981, LY-354740, MK-801, spermine, arkain). Notably, the LCGA-17 binding site was found to be distinct from those for other GABA_*A*_R ligands including isoguvacine, salicylidene salicylhydrazide, bretazenil, SL651498, MK0343, THDOC, TB21007, gaboxadol, FGIN-1-27, and allopregnanolone (Supplementary Table 1).

### Behavioral Analyses of LCGA-17 in Mice

**FIGURE 4:**
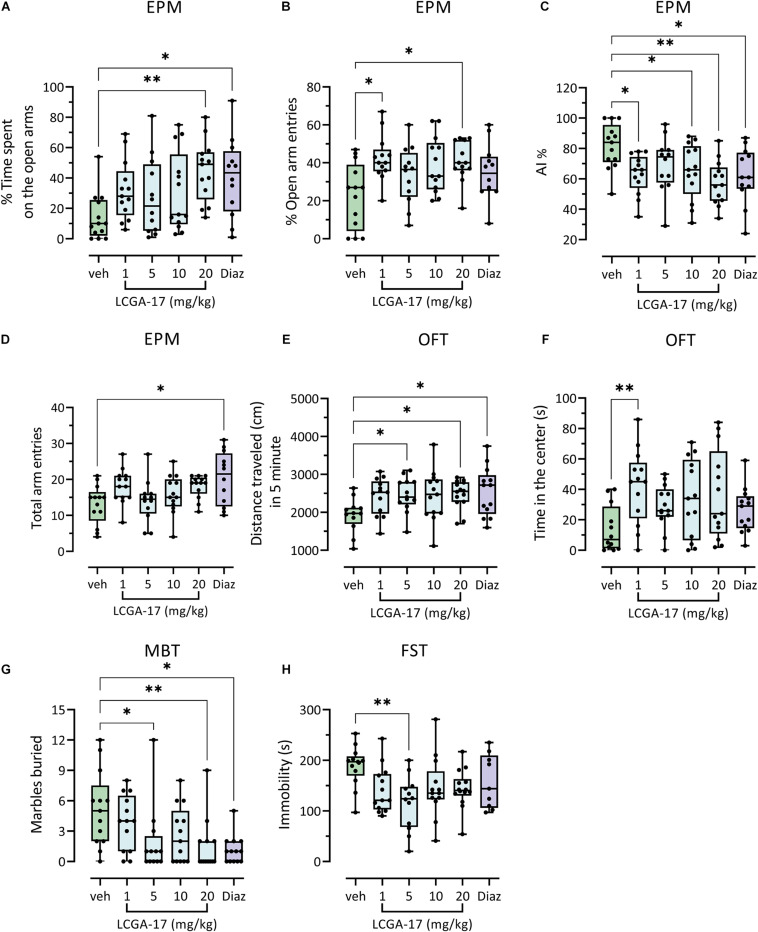
Anxiolytic- and antidepressant-like efficacy of LCGA-17 in mice. Separate groups of mice (*n* = 13) were injected with LCGA at the dosages indicated or diazepam (0.75 mg/kg) followed by behavioral analyses 30 min later, employing one test every 2–3 days. **(A–D)** In the EPM, LCGA-17 dose-dependently increased the percentage of time spent on open arms, with maximal effects at the highest dosage tested (20 mg/kg) [*H*(5, 70) = 14.9; *p* = 0.01; Kruskal–Wallis, Dunn’s test] similar to diazepam (0.75 mg/kg) **(A)**. An anxiolytic-like effect of LCGA-17 was also observed based on percent open arm entries, which was significant at both 1 and 20 mg/kg [*H*(5, 70) = 10.9; *p* = 0.05; Kruskal–Wallis, Dunn’s test] **(B)** and across the entire doses range when the two EPM parameters are combined into an Anxiety Index [*F*(5, 70) = 3.4; *p* = 0.008; ANOVA, Dunnett’s test] **(C)**. LCGA-17 had no effect on total arm entries, in contrast to diazepam [*H*(5, 70) = 12.2; *p* = 0.03; Kruskal–Wallis, Dunn’s test] **(D)**. **(E,F)** In the OFT, LCGA-17 (5, 30 mg/ml) and diazepam increased the distance traveled [*F*(5, 70) = 2.3; *p* = 0.05; ANOVA, Dunnett’s test] **(Å)**. At a dose of 1 mg/kg LCGA-17 also increased time spent in the center of the arena [*Í*(5, 70) = 11.0; *p* = 0.05; Kruskal–Wallis, Dunn’s test] **(F)**. **(G)** In the MBT, LCGA-17 (5, 20 mg/kg) reduced the number of marbles buried [*H*(5, 70) = 18.0; *p* = 0.003; Kruskal–Wallis, Dunn’s test] similar to diazepam (*p* < 0.05), indicating an anxiolytic response. **(H)** In the FST, the total time spent immobile was reduced at 5 mg/kg LCGA-17 [*F*(5, 70) = 3.1; *p* = 0.02; ANOVA, Dunnett’s test] but at none of the other dosages, nor by diazepam. ^∗^*p* < 0.05, ^∗∗^*p* < 0.01 vs. vehicle.

To explore the potential of LCGA-17 as a therapeutic in mammals we conducted dose-finding studies in mice, using a series of behavioral tests with predictive validity for anxiolytic and antidepressant drug activity in patients. In the EPM, LCGA-17 (20 mg/kg) and diazepam (0.75 mg/kg) similarly increased the percent time spent on open arms. Moreover, LCGA (unlike diazepam) had no effects on total arm entries, indicating anxiolytic-like activity without locomotor effects in this test (Figures 4A,D). An anxiolytic effect of LCGA-17 was also evident based on the increased percent time spent on open arms, which was significant at both the 1 and 20 mg/kg dosages (Figure 4B). This effect was evident across the entire dose range of the peptide (1–20 mg/kg) when the data were plotted as AI, consisting of the mean of these EPM parameters (Figure 4C). Moreover, LCGA had no effect on total arm entries, again indicating absence of locomotor effects (Figure 4D). In the OFT, LCGA-17 (5, 20 mg/ml) and diazepam modestly increased the distance traveled (Figure 4E), as previously reported for diazepam and expected as part of an anxiolytic response (Crawley, 1981). More importantly, a more overt anxiolytic response of LCGA-17 (but not diazepam) was evident based on the increased time spent in the center with all doses combined (Figure 4F and Supplementary Figure 4F) and especially significant at the lowest dose (1 mg/kg, Figure 4F). In the MBT, LCGA-17 reduced the number of marbles buried at both 5 and 20 mg/kg, thereby mimicking the anxiolytic-like effect of diazepam (Figure 4G). Collectively, these data strongly suggest that LCGA-17 has anxiolytic properties, with a rapid onset of action similar to or more potent than diazepam, yet without any sign of sedative effects even at the highest doses.

To begin to explore the potential of LCGA-17 as an antidepressant, we further subjected the mice to the FST. Although the biological underpinnings of this test are controversial, the time spent immobile in this test is known to be reduced across multiple classes of antidepressants including SSRIs, tricyclics (Detke et al., 1995), and ketamine (Ren et al., 2016), and insensitive to anxiolytic concentrations of diazepam (Detke et al., 1995). Interestingly, LCGA-17 (5 mg/kg) significantly decreased the behavioral immobility of mice (Figure 4H) and also evident when all the doses (1–20 mg/kg) were combined (Supplementary Figure 4H). Moreover, the behavioral effects of different dosages of LCGA-17 were indistinguishable from each other, for all test paradigms (for statistics see legend of Supplementary Figure 4). As predicted, diazepam failed to affect the behavior in this test. While these findings are encouraging, much more extensive experimentation will be necessary to assess the antidepressant and stress-protective potential of LCGA-17, which is beyond the scope of this initial study.

### Behavioral Effects of LCGA-17 in BIC-Treated BALB/c Mice

**FIGURE 5:**
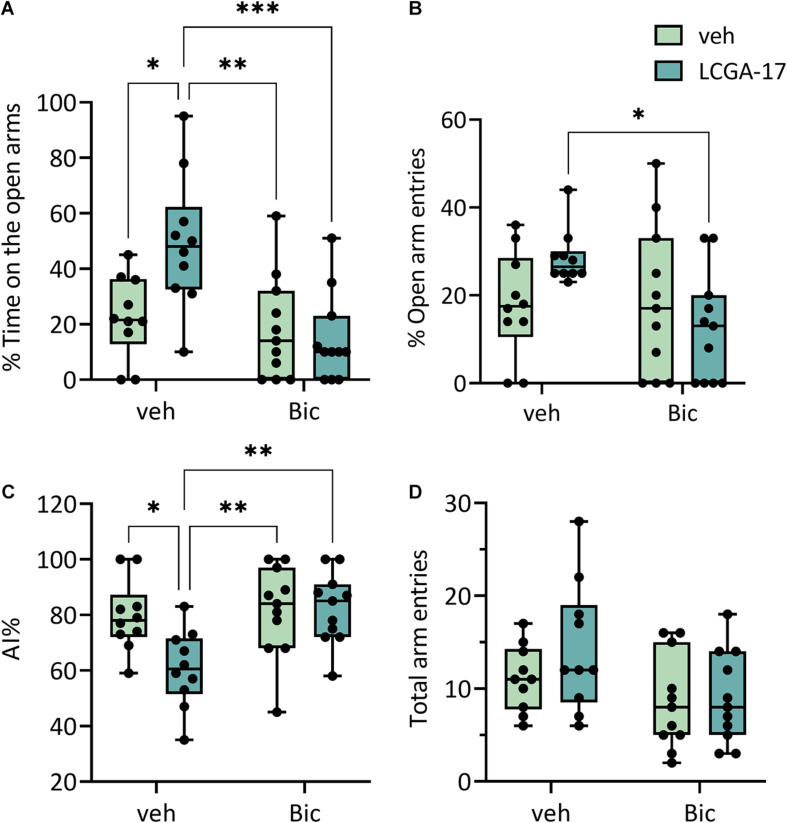
The anxiolytic effects of LCGA-17 involve enhanced GABAergic transmission. The mice (*n* = 10–11) were treated with LCGA-17 (20 mg/kg) or bicuculline (BIC, 5 mg/kg) or both, followed by analyses in the EPM. **(A)** The % time on open arms showed significant LCGA-17 [*F*(1, 38) = 4.004, *p* = 0.05] and bicuculline effects [*F*(1, 38) = 11.44, *p* = 0.002] and an LCGA-17 × bicuculline interaction [*F*(1, 38) = 0.01, *p* = 0.01]. LCGA-17 increased the % time on open arms vs. vehicle- (*p* < 0.05) and bicuculline-treated mice (*p* < 0.01). Bicuculline co-administered completely blocked the effect of LCGA-17 [Bic + LCGA-17 vs. LCGA-17, *p* < 0.001; LCGA-17 + Bic vs. veh, *p* (ns)]. By contrast, bicuculline alone had no effect. **(B)** The number of open arm entries showed an LCGA-17 × bicuculline interaction [*F*(1, 38) = 4.58, *p* = 0.039] and bicuculline reduced the LCGA-17 effect (LCGA-17 + Bic vs. LCGA-17, *p* < 0.05), again without effects of bicuculline alone. **(C)** The AI showed LCGA [*F*(1, 38) = 4.38, *p* = 0.045] and bicuculline main effects [*F*(1, 38) = 7.34, *p* = 0.01] and an LCGA-17 × bicuculline interaction [*F*(1, 38) = 5.12, *p* = 0.029]. LCGA-17 reduced the AI compared to vehicle- (LCGA-17 vs. veh, *p* < 0.05) and bicuculline-treated mice (LCGA-17 vs. Bic, *p* < 0.01). This anxiolytic effect of LCGA-17 was reversed by cotreatment with bicuculline [LCGA-17 vs. LCGA-17 + Bic, *p* < 0.01; veh vs. LCGA-17 + Bic, *p* (ns)]. **(D)** Bicuculline slightly reduced the total arm entries in this test [*F*(1, 38), *p* = 0.022] but *post hoc* pairwise group comparisons were not significant. ^∗^*p* < 0.05, ^∗∗^*p* < 0.01, ^∗∗∗^*p* < 0.001; ANOVA, Tukey’s test.

The rapid anxiolytic actions of gabapentinoids are thought to involve increased GABA_*A*_R activity, reminiscent of diazepam but mediated by increased cell surface expression instead of direct activation of GABA_*A*_Rs (Yu et al., 2019). Therefore, to begin to test whether the behavioral effects of LCGA-17 in the EPM involved enhanced GABAergic transmission, we co-treated the mice with LCGA-17 and the GABA_*A*_R antagonist bicuculline. Interestingly, the LCGA-17-induced increase in the percent time on open arms was completely abolished by bicuculline, without effects of bicuculline alone (Figure 5A). Similarly, co-treatment of mice with LCGA-17 and bicuculline reduced the % Open Arm Entries compared to the effect of LCGA-17 alone (Figure 5B) (for ANOVA statistics see Figure legend). Lastly, bicuculline blocked the effect of LCGA-17 on the AI, again without effects of bicuculline alone (Figure 5C) and without effects on locomotion (Figure 5D). In summary, LCGA-17 has been identified as a novel rapid-acting anxiolytic and potential antidepressant that acts via α2δ and possibly GABA_*A*_Rs to enhance ionic GABAergic neurotransmission.

## Discussion

We here describe the identification of LCGA-17 as a novel, CNS-active peptide with anxiolytic- and possibly antidepressant-like properties in mice. LCGA-17 was selected from a digital library of milk casein-derived tetrapeptides, based on its ability to dock to GABA_*A*_Rs and α2δ *in silico*, and through a high throughput screen in *D. rerio* for anxiolytic-like behavioral efficacy. Further characterization in mice confirmed that LCGA-17 has potent anxiolytic-like effects in multiple behavioral tests, with additional potential as an antidepressant.

We aimed our computational strategy at a dual-acting compound since positive modulators of GABA_*A*_Rs and inhibitors of α2δ have well established overlapping therapeutic applications. Moreover, it so happened that LCGA-17 showed evidence for appreciable affinity for both proteins, with Autodock Vina binding energies of −7.8 kcal/mol for the putative GABA_*A*_ PREGS binding pocket and −8.5 kcal/mol for the gabapentin binding pocket of α2δ, respectively. We note, however, that the high binding energy for GABA_*A*_Rs was observed specifically with LCGA-17 docked to the “narrow-lumen” conformation of α1β2γ2 GABA_*A*_Rs, while the “wide-lumen” conformation of this receptor predicted a significantly lower affinity (ΔG, −7.0 kcal/mol). This difference in binding energy is reflected in different attractive interactions formed by the two receptor structures (Figures 1B,D). The lower-affinity, “wide-lumen” receptor conformation was largely devoid of hydrogen bonding and attractive electrostatic interactions with LCGA-17, implying that binding to the receptor relies mainly on hydrophobic interactions that are unlikely effective to influence channel structure and function. Notably and independent of the two binding pockets of the two GABA_*A*_R conformations, LCGA-17 adopted a highly distinctive structure resembling a cork plugged into the channel. Given the mode of interaction above we were not surprised to find that LCGA-17 lacked appreciable affinity for GABA_*A*_Rs in radioligand binding assays (IC_50_ > 100 μM). Electrophysiological characterization in combination with site-directed mutagenesis of GABA_*A*_Rs will be required to further examine the functional relevance of GABA_*A*_Rs as a direct target of LCGA-17.

Compared to GABA_*A*_Rs, much less is known about structure-function relationships of α2δ. This is illustrated by the fact that gabapentin, the most widely known ligand of α2δ, to this day lacks a well-defined binding site. By contrast, our modeling experiments revealed a likely α2δ binding pocket for LCGA-17. Binding was found to be dynamically stable and supported by numerous putative molecular interactions that result in a high binding energy (−8.5 kcal/mol). Consistent with these modeling results, radioligand binding assay using cortical brain membranes identified a binding site for [^3^H]-LCGA-17 of low micromolar affinity (IC_50_ = 2 μM) that was effectively competed also by gabapentin (IC_50_ = 11 μM). By contrast PREGS failed to compete, which confirmed α2δ rather than GABA_*A*_Rs as the primary interaction partner. Lastly, no cross-reactivity was observed between the [^3^H]-LCGA-17 binding site and a library of known ligands for other major neurotransmitter receptors (Supplementary Table 1). Collectively, these observations strongly suggest that α2δ serves as the primary target of LCGA-17.

Effective competition of [^3^H]-LCGA-17 by gabapentin suggested moderate affinity for a putative gabapentin and pregabalin binding site, identified by modeling, that mapped to the same binding pocket of LCGA-17 on α2δ. However, our modeling experiments revealed several additional putative α2δ binding sites for gabapentinoids, indicating that modeling alone is insufficient to unambiguously determine the most relevant among these sites. Similarly, we cannot currently exclude the existence of other, unrelated, and even higher affinity targets for LCGA-17. Saturation assays will be necessary to assess whether the gabapentin binding site of α2δ is the primary or merely one of several candidate targets for LCGA-17. Lastly, binding assays alone are insufficient to predict functional efficacy, which will require electrophysiological analyses of targets in a heterologous expression system, preferably combined with mutagenesis of putative target sites.

Target site predictions for LCGA-17 are further complicated by the dependency of α2δ on multiple, functionally distinct effector ion channels. In particular, recent evidence suggests that the anxiolytic and antinociceptive mechanisms of gabapentin are mechanistically independent and mediated by distinct indirect ion channel targets. While the antinociceptive effects of gabapentin involve inhibition and normalization of α2δ-dependent cell surface trafficking of axonal NMDA receptors (Chen et al., 2018; Shi et al., 2018; Deng et al., 2019), its anxiolytic behavioral effects involve rapidly increased tonic inhibition of neurons by δ subunit-containing GABA_*A*_Rs (Yu et al., 2019). This latter mechanism is relevant for LCGA-17 because its anxiolytic activity was effectively blocked by the GABA_*A*_R antagonist bicuculline. Therefore, the interaction of LCGA-17 with α2δ together with its rapid anxiolytic effect in mice and the sensitivity of these behavioral effects to bicuculline raise the possibility that δ-GABA_*A*_Rs serve as yet an additional target of α2δ and/or that LCGA-17 functions in a manner analogous to gabapentin through a not yet identified target to ultimately increase GABAergic tonic inhibition.

Our behavioral characterization of LCGA-17 in mice revealed rapid and robust anxiolytic-like effects in three independent behavioral tests. These effects were observed over a wide range of doses (1–20 mg/kg), suggesting therapeutic efficacy comparable or better than diazepam, and they came without any sign of sedation even at the highest dose. In particular, LCGA-17 was more potent than diazepam as an anxiolytic in the OFT. Lastly, in the FST, LCGA-17 mimicked the anti-despair-like effects of chronic treatment with SSRIs and acute doses of subanesthetic ketamine, suggesting that LCGA-17 has potential as a rapid acting antidepressant. Diazepam was inactive in this test, which is in keeping with previous reports (Martí and Armario, 1993) and at best limited efficacy of benzodiazepines as antidepressants (Benasi et al., 2018). A LCGA-17 mechanism involving gabapentin-like increases in GABAergic inhibition would be consistent with evidence that depressive disorders are caused by defects in GABAergic neural inhibition (Luscher and Fuchs, 2015) and efficacy of pregnanolone and zuranolone as rapid acting GABAergic antidepressants (Lüscher and Möhler, 2019; Althaus et al., 2020). Notably, the anxiolytic-like and acute stress-relieving properties of LCGA-17 were also evident in fish, which suggests evolutionarily conservation of the behaviorally relevant target(s) of LCGA-17 across most if not all vertebrates.

Our experiments revealed a lack of dose dependency of LCGA-17 induced behavioral responses, which seems inconsistent with a relatively low affinity interaction of LCGA-17 with its putative target(s) observed *in vitro*. Curiously, our preliminary and ongoing pharmacokinetic studies indicate a short *in vivo* half-life for LCGA-17 (minutes), yet the behavioral effects in rodents last for hours if not days. Future studies will need to assess whether LCGA-17 induces a ketamine-like, rapid mechanism of neural plasticity (Deyama and Duman, 2020; Luscher et al., 2020), that would explain the limited dose dependency of LCGA-treatment and behavioral responses at a time when the drug is no longer on board.

## Conclusion

In conclusion, LCGA-17 is a novel rapid acting anxiolytic with potential antidepressant properties that binds with significant affinity to a gabapentin binding site on α2δ. Its anxiolytic properties are sensitive to bicuculline, which predicts a mechanism similar to gabapentin-mediated, indirect enhancement of tonic GABAergic inhibition. Additionally, LCGA-17 may interact directly with GABA_*A*_Rs at the PREGS binding site or with other, so far unknown targets. Additional characterization of LCGA-17 will be necessary to ascertain its direct and indirect targets as well as its cellular mechanism and therapeutic potential as an antidepressant.

## Data Availability Statement

The raw data supporting the conclusions of this article will be made available by the authors, without undue reservation.

## Ethics Statement

The animal study was reviewed and approved by Bioethics Commission LLC of the Institute of Mitoengineering of MSU [protocols No. 118 (31.05.2018) and No. 134 (13.05.2019).

## Author Contributions

AM and BL: conceptualization. ASZ, AOZ, VG, VP, and EV: investigation. AG, ML, GK, and YZ: methodology. AM, ID, NM, and AK: project administration. ASZ and AOZ: software. GB, AG, GK, and YZ: supervision. IS and ASZ: visualization. IS and ASZ: roles/writing—original draft. BL, AM, IS, and ASZ: writing-review editing. All authors contributed to the article and approved the submitted version.

## Conflict of Interest

AM, IS, ASZ, VG, VP, ID, NM, AK, GB are employed by Lactocore, Inc. BL is a consultant for Lactocore, Inc. The remaining authors declare that the research was conducted in the absence of any commercial or financial relationships that could be construed as a potential conflict of interest.

## Publisher’s Note

All claims expressed in this article are solely those of the authors and do not necessarily represent those of their affiliated organizations, or those of the publisher, the editors and the reviewers. Any product that may be evaluated in this article, or claim that may be made by its manufacturer, is not guaranteed or endorsed by the publisher.

## Abbreviations

5-HT: serotonin
AChR: acetylcholine receptor
Bic: bicuculline
BZD: benzodiazepine
CNS: central nervous system
DA: dopamine
EPM: elevated plus maze
Diaz: diazepam
FST: forced swim test
Flv: Fluvoxamine
GABA: γ -aminobutyric acid
GABA_*A*_: γ -aminobutyric acid type A
GAD: general anxiety disorder
HSCIE: high-temperature solid-phase catalytic isotope exchange
IC_50_: half-maximal inhibitory concentration
i.p.: intraperitoneal
LCMS/MS: liquid chromatography-tandem mass spectrometry
LDB: light–dark box
MBT: marble-burying test
MD: molecular dynamics
MDD: major depressive disorder
NMDA: *N*-methyl -D-aspartate
NTT: novel tank test
OFT: open field test
PAM: positive allosteric modulator
SPT: shoaling preference test
SSRI: selective serotonin reuptake inhibitor
VGCC: voltage-gated calcium channel.
