# Supplementary material for: In silico Screening and Behavioral Validation of a Novel Peptide, LCGA-17, With Anxiolytic-Like Properties
Source: Front Neurosci. 2021 Aug 2;15:705590. doi: 10.3389/fnins.2021.705590 (PMC8372404; doi:10.3389/fnins.2021.705590)
Supplement: Supplementary file 1 [file Data_Sheet_1.docx]

**Supplementary Materials and Methods**

**Molecular Dynamics Simulation Protocol**

All MD simulations were performed with Gromacs 2020 software (Abraham et al., 2015). All models discussed were initially preprocessed with MolProbity to correct protonation states, amide rotamers, and histidine tautomers (Williams et al., 2018). Unliganded and liganded ɑ2δ-1 VGCC systems were subjected to an initial 50,000 step minimization and filled with TIP3P water molecules, and the total charge was neutralized with 0.15 M Na^+^ or Cl^-^ ions. Then another minimization was performed. 100 ps of each positional-restrained NVT and NPT equilibration were carried out with subsequent 10ns unrestrained NPT equilibration. All equilibration and production simulations were performed with 2fs timestep, 300K temperature, NPT simulations under 1-atmosphere pressure. Velocity rescale algorithm was used to control the temperature while pressure coupling was controlled by Berendsen barostat in the equilibration phase and Parrinello-Rahman barostat in production (Parrinello and Rahman, 1981; Berendsen et al., 1984; Bussi et al., 2007). In both equilibration and production simulations of the unliganded system the backbone of initially resolved residues was positionally restrained by a 1000 kJ/mol/nm^2 potential. Additionally, the reconstructed loop was thermally coupled separately from the rest of the protein and the solution, with reference temperatures of 400K, 200K, and 300K respectively. The complex of ɑ2δ-1 and FQSE peptide was not positionally restrained during the production run, and thermal coupling was performed separately for the whole protein versus non-protein entities with both reference temperatures of 300K. Both liganded and unliganded systems were modeled with the amber99sb-ildn force field (Lindorff-Larsen et al., 2010).

GABA_A_R in complex with FQSE peptide was inserted into the membrane model and solvated with CHARMM-GUI web server (Jo et al., 2008). After the default minimization and equilibration protocol on the server-side, inputs for production simulation in Gromacs were obtained (Lee et al., 2016). 100ns production run was performed with Velocity rescale thermostat and Parrinello-Rahman barostat in semi-isotropic setup.

# **Supplementary Figures**

*
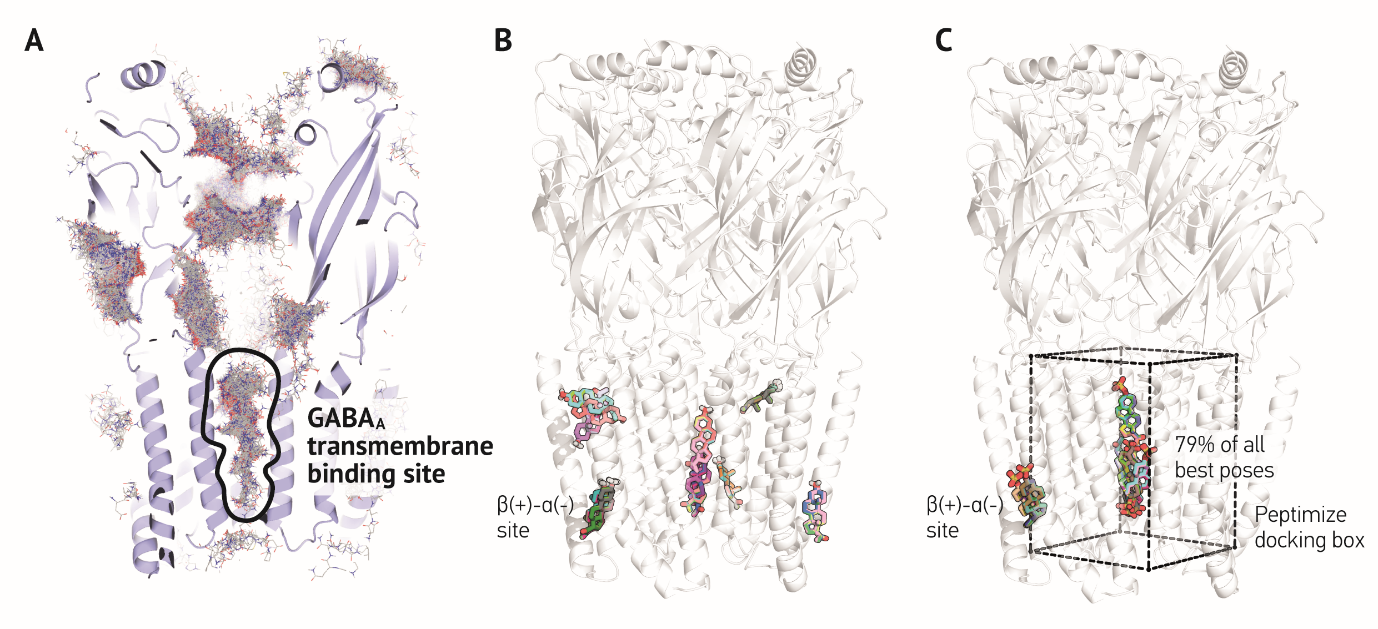
*

*Figure S1.* **Identification of a GABA_A_ receptor binding site of interest. A.** Spatial clustering of best docking poses of the hydrolysate peptides in the ɑ1β2γ2 GABA_A_R models. The transmembrane domains of four GABA_A_R subunits are shown as α-helical structures with the transmembrane binding pocket encircled in black. **B.** Spatial clustering of allopregnanolone with the best docking poses of the ɑ1β2γ2 GABA_A_R models. **C.** Spatial clustering of the best docking poses for pregnenolone sulfate in the ɑ1β2γ2 GABA_A_ receptor models. Black dashes indicate the docking box used for subsequent Peptimize calculations.

*
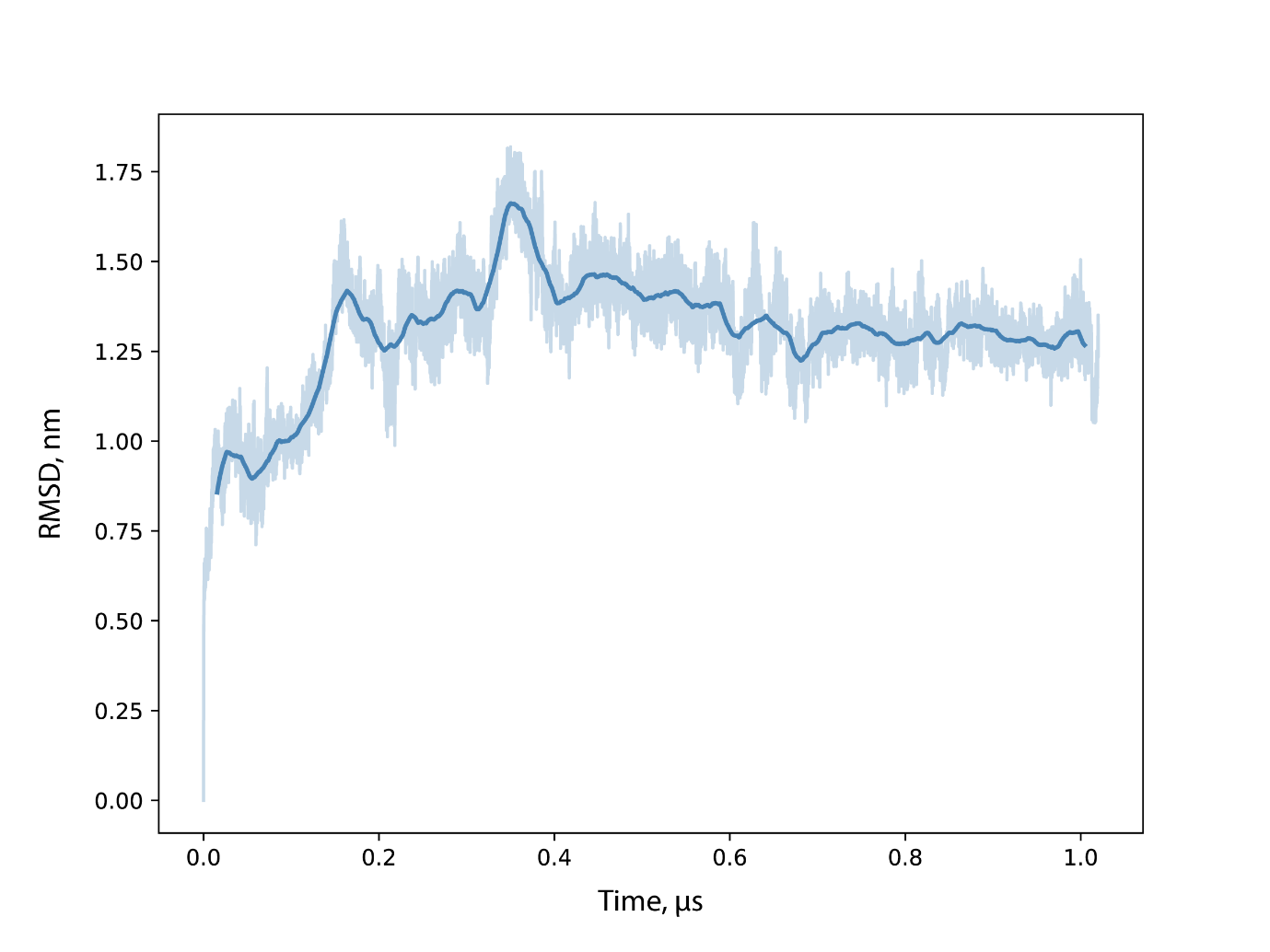
*

*Figure S2.* **Dynamics of the reconstructed Pro913-Met972** **loop of ɑ2δ-1 VGCC.** The solid line represents the moving average of a 40 ns window.

*
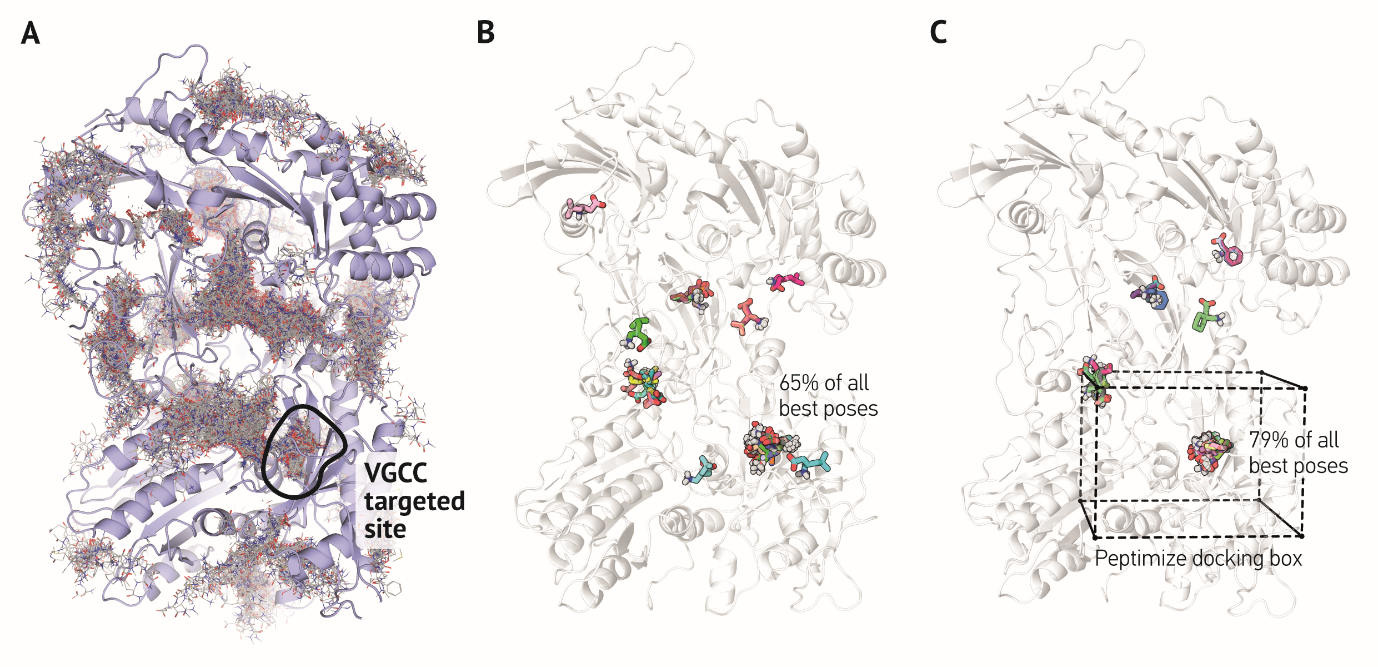
*

*Figure S3.* **Identification of an ɑ2δ-1 VGCC binding site. A.** Spatial clustering of the best docking poses for the hydrolysate peptides against the 700 ns ɑ2δ-1 model. **B.** Spatial clustering of the best docking poses for pregabalin on the 700 ns ɑ2δ-1 model. **C.** Spatial clustering of the best docking poses for gabapentin on ɑ2δ-1 VGCC. Black dashes indicate the docking box used for subsequent Peptimize calculations.

*
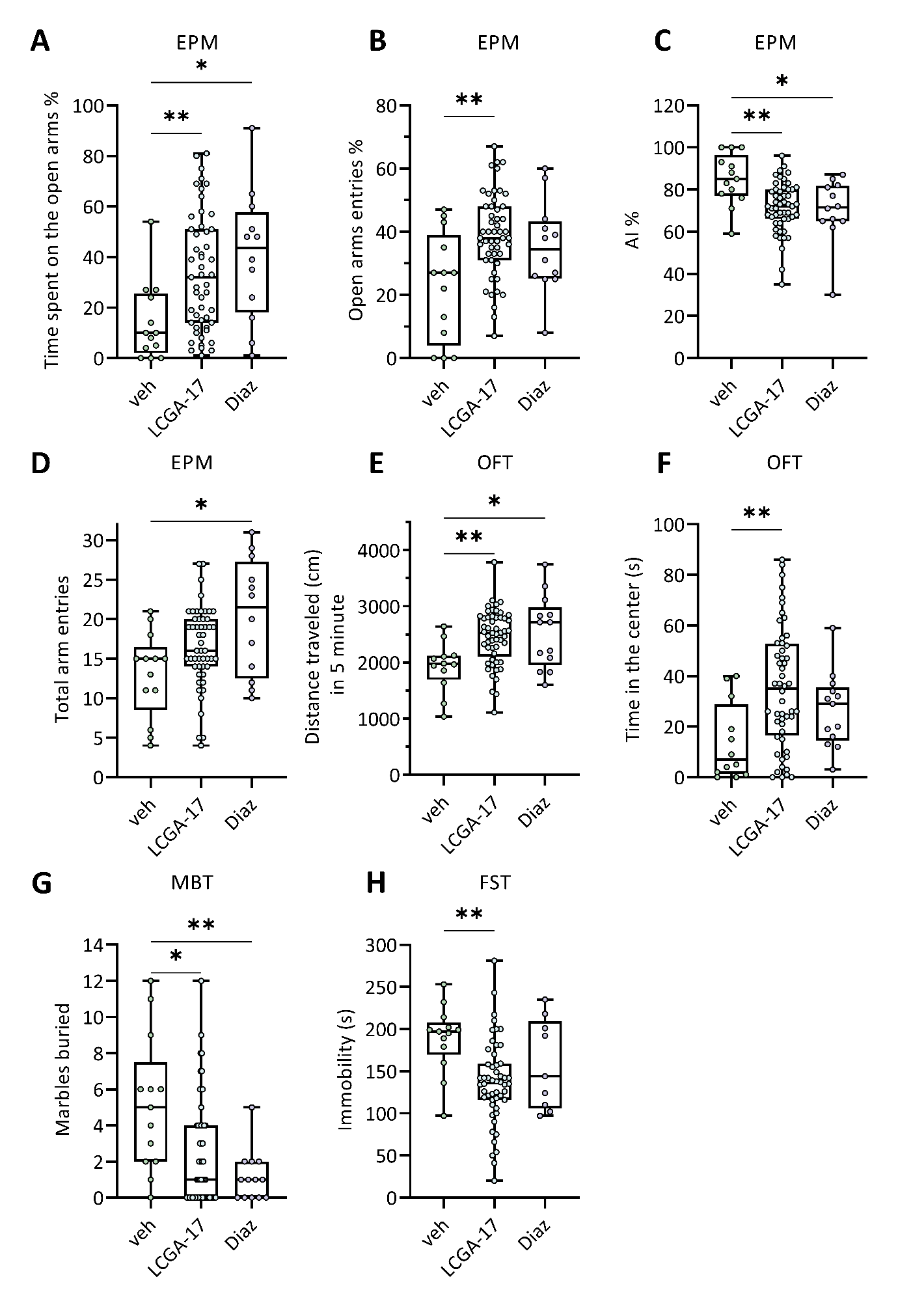
*

*Figure S4.* **Anxiolytic- and antidepressant-like efficacy of LCGA-17 in mice with the different LCGA-17-treatment groups (1, 5, 10, and 20 mg/kg, n = 13 each) combined.** **A-D**) In the EPM, LCGA-17 increased the percentage of time spent on open arms [H(2, 73) = 10.2; p = 0.006] similar to diazepam (p = 0.01, 0.75 mg/kg)(A). An anxiolytic-like effect of LCGA-17 was also observed based on percent open arm entries [H(2, 73) = 8.3; p = 0.001](B) and Anxiety Index [H(2, 73) = 10.5; p = 0.005](C). LCGA-17 had no effect on total arm entries, in contrast to diazepam [H(2, 73) = 6.8; p = 0.03](D). **E, F**) In the OFT, LCGA-17 and diazepam increased the distance traveled [H(2, 73) = 10.0; p = 0.007](Е). LCGA-17 also increased time spent in the center of the arena [Н(2, 73)= 9.2; p=0.01](F). **G**) In the MBT, LCGA-17 reduced the number of marbles buried similar to diazepam, indicating an anxiolytic response [H(2, 73)=10.0; p = 0.007]. **H**) In the FST, the total time spent immobile was reduced in the LCGA-17 group [H(2, 73) = 10.6; p = 0.005], but not in diazepam. *p < 0.05, **p < 0.01 vs. vehicle; Kruskal-Wallis, Dunn’s test.

Before combining data across all LCGA-17 groups, we have checked the effect of dosage on behavioral response in mice. In the EPM, no effect of dose was found on the percent of time spent on open arms [H(3, 48) = 4.97; p = 0.17; Kruskal-Wallis], percent open arm entries [H(3, 48) = 2.66; p = 0.45; Kruskal-Wallis], total arm entries[H(3, 48) = 6.46; p = 0.09; Kruskal-Wallis], and Anxiety Index [H(3, 48) = 4.19; p = 0.24; Kruskal-Wallis]. In the OFT, no effect of LCGA-17 dose was found on distance traveled [F(3, 48) = 0.03; p = 0.99; one-way ANOVA], and time spent in the center [H(3, 48) = 1.96; p = 0.58; Kruskal-Wallis]. In the MBT, the effect of dose was also absent [H(3, 48) = 7.15; p = 0.07; Kruskal-Wallis]. In the FST, the total time spent immobile did not differ across dosages of LCGA-17 [F(3, 48) = 1.01; p = 0.39; one-way ANOVA].

## **Supplementary videos**

**Supplementary video 1**. Stabilization of LCGA-17 peptide binding mode within the transmembrane domain of the wider-lumen conformation of GABA-A receptor.

**Supplementary video 2**. Stabilization of LCGA-17 peptide binding mode within the transmembrane domain of the narrower-lumen conformation of GABA-A receptor.

**Supplementary video 3**. Stabilization of LCGA-17 peptide binding mode on the a2d domain of VGCC receptor.

**References**

Abraham, M. J., Murtola, T., Schulz, R., Páll, S., Smith, J. C., Hess, B., et al. (2015). Gromacs: High performance molecular simulations through multi-level parallelism from laptops to supercomputers. *SoftwareX* 1–2, 19–25. doi:10.1016/j.softx.2015.06.001.

Berendsen, H. J. C., Postma, J. P. M., Van Gunsteren, W. F., Dinola, A., and Haak, J. R. (1984). Molecular dynamics with coupling to an external bath. *The Journal of Chemical Physics* 81, 3684–3690. doi:10.1063/1.448118.

Bussi, G., Donadio, D., and Parrinello, M. (2007). Canonical sampling through velocity rescaling. *Journal of Chemical Physics* 126, 014101. doi:10.1063/1.2408420.

Jo, S., Kim, T., Iyer, V. G., and Im, W. (2008). CHARMM-GUI: A web-based graphical user interface for CHARMM. *Journal of Computational Chemistry* 29, 1859–1865. doi:10.1002/jcc.20945.

Lee, J., Cheng, X., Swails, J. M., Yeom, M. S., Eastman, P. K., Lemkul, J. A., et al. (2016). CHARMM-GUI Input Generator for NAMD, GROMACS, AMBER, OpenMM, and CHARMM/OpenMM Simulations Using the CHARMM36 Additive Force Field. *Journal of Chemical Theory and Computation* 12, 405–413. doi:10.1021/acs.jctc.5b00935.

Lindorff-Larsen, K., Piana, S., Palmo, K., Maragakis, P., Klepeis, J. L., Dror, R. O., et al. (2010). Improved side-chain torsion potentials for the Amber ff99SB protein force field. *Proteins: Structure, Function and Bioinformatics* 78, 1950–1958. doi:10.1002/prot.22711.

Parrinello, M., and Rahman, A. (1981). Polymorphic transitions in single crystals: A new molecular dynamics method. *Journal of Applied Physics* 52, 7182–7190. doi:10.1063/1.328693.

Williams, C. J., Headd, J. J., Moriarty, N. W., Prisant, M. G., Videau, L. L., Deis, L. N., et al. (2018). MolProbity: More and better reference data for improved all-atom structure validation. *Protein Science* 27, 293–315. doi:10.1002/pro.3330.
