# Supplementary material for: In silico Screening and Behavioral Validation of a Novel Peptide, LCGA-17, With Anxiolytic-Like Properties
Source: Front Neurosci. 2021 Aug 2;15:705590. doi: 10.3389/fnins.2021.705590 (PMC8372404; doi:10.3389/fnins.2021.705590)
Supplement: Supplementary file 2 [file Table_1.DOCX]

**Table S1. LCGA-17 competitors tested in radioligand binding assays**^1^ Sigma Aldrich; ^2^ Gedeon Richter; ^3^ Fluka; ^4^ Tocris Bioscience; ^5^ Axon; ^6^ Santa Cruz

| Studied compound | Target receptor/ion channel | IC_50_ (µM) |
| --- | --- | --- |
| 7-OH-DPAT ^1^ | Dopamine D3R agonist | >100 |
| Haloperidol ^2^ | Dopamine D2,3,4R antagonist; Sigma1 R ligand. | >100 |
| Spiperone ^1^ | D2/D4R antagonist; α_1B_-adrenoceptor antagonist; 5-HT2a/5-HT1R antagonist | >100 |
| Sulpiride ^1^ | Dopamine D2,3R and 5-HT1AR antagonist | >100 |
| Ketanserin ^1^ | 5HT2aR antagonist | >100 |
| Bicuculline ^3^ | GABA_A_R competitive antagonist | >100 |
| GABA ^1^ | GABA_A_R and GABA_B_R agonist | >100 |
| Allopregnanolone ^4^ | GABA_A_R positive allosteric modulator | >100 |
| Bretazenil ^4^ | GABA_A_R BZD site α1 subtype-selective agonist | >100 |
| CGS-9895 ^1^ | GABA_A_R BZD site antagonist | >100 |
| Diazepam ^1^ | GABA_A_R BZD site agonist | >100 |
| Flumazenil ^1^ | GABA_A_R BZD site antagonist | >100 |
| Gaboxadol (THIP) ^4^ | GABA_A_R α4β3δ subtype partial agonist | >100 |
| MK0343 ^4^ | GABA_A_R α3 subtype-selective agonist | >100 |
| Muscimol ^3^ | GABA_A_R agonist | >100 |
| Pregnenolone ^1^ | GABA_A_R negative allosteric modulator | >100 |
| [Salicylidene salicylhydrazide](https://pubmed.ncbi.nlm.nih.gov/15100159/) ^4^ | GABA_A_R antagonist | >100 |
| SL 651498 ^5^ | GABA_A_R α2 subtype-selective agonist | >100 |
| Gabazine ^6^ | GABA_A_R antagonist selective for extrasynaptic receptors | >100 |
| TB21007 ^4^ | GABA_A_R α5 subtype-selective inverse agonist | >100 |
| THDOC ^4^ | GABA_A_R α4,6 subtype-selective agonist | >100 |
| Zolpidem ^1^ | GABA_A_R BZD site agonist (α_1s_subtype-selective) | >100 |
| Baclofen ^1^ | GABA_B_R agonist | >100 |
| Glutamate ^1^ | Glutamate receptor agonist | >100 |
| Arcaine ^1^ | NMDAR polyamine site ligand | >100 |
| Spermine ^1^ | NMDAR polyamine site ligand | >100 |
| Glycine ^1^ | NMDAR glycine site agonist | >100 |
| Ifenprodil ^6^ | NMDAR inhibitor | >100 |
| MK-801 ^1^ | NMDAR noncompetitive antagonist | >100 |
| Ro-256981 ^6^ | NMDAR GluN2B subunit selective antagonist | >100 |
| LY-354740 ^6^ | mGlu2R-selective agonist | >100 |
| FGIN-1-27 ^4^ | Mitochondrial diazepam-binding inhibitor receptor ligand | >100 |
| Gabapentin ^1^ | α2δ subunit-containing VGCCs, antagonist | **11 ± 0.1** |
| GBR-12909 ^4^ | Dopamine reuptake inhibitor | >100 |
| Nicotine ^4^ | Nicotinic AChR agonist | >100 |
| PK-11195 ^1^ | TSPO (translocator protein, peripheral BZD receptor ligand) | >100 |
