# Supplementary material for: In silico Screening and Behavioral Validation of a Novel Peptide, LCGA-17, With Anxiolytic-Like Properties
Source: Front Neurosci. 2021 Aug 2;15:705590. doi: 10.3389/fnins.2021.705590 (PMC8372404; doi:10.3389/fnins.2021.705590)
Supplement: Supplementary file 3 [file Table_2.DOCX]

**Table S2 Behavioral Analyses of *Danio rerio* in the NTT, LDB and SPT.** Descriptive statistics (median, Q1 – lower, Q3 – upper quartiles) and pairwise comparison (M-W p value) of drug-treated groups (exp) vs. vehicle-treated groups (veh).

|  |  | NTT. Time spent in  upper half of tank (s) | | | NTT. Distance traveled (cm) | | | LDB. Time spent in  lit compartment (s) | | | SPT. Time spent out of  shoaling zone (s) | | |
| --- | --- | --- | --- | --- | --- | --- | --- | --- | --- | --- | --- | --- | --- |
| **Treatment** | **group** | Median | Q1; Q3 | **p value** | Median | Q1; Q3 | **p value** | **Median** | **Q1; Q3** | **p value** | **Median** | **Q1; Q3** | **p value** |
| diaz  1.25 mg/kg | veh | 0.7 | (0; 22) | ***0.006*** | 941 | (199; 1640) | 0.934 | 73 | (16; 193) | 0.077 | 3 | (0; 34) | ***0.028*** |
|  | exp | 0.0 | (0; 0.4) |  | 887 | (183; 1394) |  | 21 | (3; 114) |  | 20 | (3; 33) |  |
| diaz  5 mg/kg | veh | 26 | (0; 75) | ***0.001*** | 1007 | (498; 1807) | 0.627 | 19 | (3; 118) | ***0.004*** | 4 | (0.4; 11) | ***0.0005*** |
|  | exp | 0.0 | (0; 2.2) |  | 595 | (326; 2079) |  | 177 | (6; 300) |  | 21 | (9; 93) |  |
| flv  5 mg/kg | veh | 16 | (1; 42) | ***0.042*** | 1623 | (746; 2166) | 0.294 | 122 | (78; 239) | 0.435 | 56 | (25; 108) | 0.135 |
|  | exp | 41 | (9; 70) |  | 1267 | (739; 1939) |  | 118 | (37; 292) |  | 90 | (45; 187) |  |
| flv  10 mg/kg | veh | 0.6 | (0; 23) | ***0.018*** | 1208 | (627; 2050) | 0.692 | 72 | (6; 266) | 0.327 | 63 | (19; 97) | 0.113 |
|  | exp | 32 | (1.6; 45) |  | 1313 | (414;1820) |  | 94 | (11; 281) |  | 84 | (53; 109) |  |
| LCGA-17 1 mg/kg | veh | 68 | (58; 77) | 0.084 | 1573 | (1315; 1983) | 0.729 | 199 | (102; 246) | 0.432 | 55 | (21; 94) | 0.268 |
|  | exp | 57 | (44; 63) |  | 1613 | (1366;1783) |  | 89 | (45; 251) |  | 39 | (14; 72) |  |
| LCGA-17  10 mg/kg | veh | 54 | (42; 74) | ***0.004*** | 1491 | (1247; 1565) | 0.141 | 66 | (22; 141) | ***0.006*** | 10 | (3; 19) | ***0.006*** |
|  | exp | 80 | (67; 87) |  | 1596 | (1360;1861) |  | 208 | (78; 252) |  | 31 | (6; 60) |  |
| LCGA-26  1 mg/kg | veh | 57 | (43; 82) | 0.346 | 1298 | (896;1605) | 0.713 | 220 | (134; 246) | 0.317 | 6 | (1; 20) | ***0.020*** |
|  | exp | 58 | (31; 78) |  | 1365 | (797; 1872) |  | 215 | (62; 259) |  | 20 | (8; 48) |  |
| LCGA-26  10 mg/kg | veh | 66 | (56; 83) | 0.269 | 1329 | (1069; 1763) | 0.519 | 208 | (129; 279) | 0.312 | 31 | (7; 97) | 0.138 |
|  | exp | 72 | (61; 93) |  | 1445 | (1190; 1766) |  | 243 | (137; 274) |  | 27 | (5; 46) |  |
| LCGA-59  1 mg/kg | veh | 59 | (28; 102) | 0.210 | 1470 | (506; 1941) | 0.350 | 84 | (50; 149) | 0.219 | 55 | (30; 115) | 0.323 |
|  | exp | 69 | (56; 95) |  | 1730 | (1311; 2127) |  | 74 | (27; 167) |  | 52 | (32; 77) |  |
| LCGA-59  10 mg/kg | veh | 83 | (68; 101) | 0.200 | 1897 | (1163; 2296) | 0.993 | 79 | (35; 123) | 0.191 | 51 | (48; 105) | 0.293 |
|  | exp | 74 | (51; 97) |  | 1636 | (1360; 2036) |  | 87 | (43; 234) |  | 88 | (41; 121) |  |
| LCGA-83  1 mg/kg | veh | 55 | (46; 73) | 0.274 | 1358 | (1199; 1641) | 0.516 | 247 | (104; 274) | 0.124 | 24 | (4; 67) | 0.238 |
|  | exp | 73 | (49; 88) |  | 1500 | (1145; 1765) |  | 128 | (87; 228) |  | 37 | (11; 63) |  |
| LCGA-83  10 mg/kg | veh | 61 | (47; 103) | 0.394 | 1271 | (1099; 1335) | 0.082 | 260 | (176; 278) | 0.318 | 52 | (18; 81) | 0.475 |
|  | exp | 76 | (52; 87) |  | 1589 | (1126; 1883) |  | 249 | (185;268) |  | 46 | (19; 91) |  |
